# The Effect of Serum Carnosinase on the Tissue Distribution of Imidazole Dipeptides After Their Oral Administration in Golden Hamsters

**DOI:** 10.3390/nu18060999

**Published:** 2026-03-21

**Authors:** Shigenobu Shiotani, Takumi Kawashima, Chikako Takahashi, Taiken Sakano, Ayumu Kuramoto, Nobuya Yanai

**Affiliations:** Food Research Institute, Tokai Bussan Co., Ltd., Tokyo 101-0032, Japan; kawashima@tokaibsn.co.jp (T.K.); c-takahashi@tokaibsn.co.jp (C.T.); sakano@tokaibsn.co.jp (T.S.); kuramoto@tokaibsn.co.jp (A.K.)

**Keywords:** carnosinase, imidazole dipeptide, anserine, carnosine, Nπ-methyl-histidine, golden hamster

## Abstract

**Background/Objectives**: Imidazole dipeptides (IDPs), carnosine and anserine, are endogenous antioxidants. The metabolism and functions of IDPs have mainly been investigated in rodents. However, the blood of primates, such as humans, contains carnosinase (CN1), which hydrolyzes IDPs. In non-primates, CN1 is absent, allowing IDPs to be distributed throughout tissues. There are concerns about whether the results of animal experiments can be directly applied to humans. Therefore, we aimed to investigate the blood change in the concentration and tissue distribution of IDPs following their oral administration to golden hamsters, the only non-primates known to possess CN1. **Methods**: Plasma CN1 activity was compared between hamsters and humans. Hamsters were administered IDPs (an anserine/carnosine mixture) purified from chicken meat at a dose of 1000 mg/kg. Blood samples were collected at time points up to 6 h after administration. Tissue samples were collected at 6 h after administration to measure the concentrations of IDPs and related substances. Additionally, IDP levels in human and mice tissues from previous studies were compared with that of hamster tissues in this study. **Results**: Hamster plasma CN1 activity was more than 10 times higher than that in humans. Although IDPs were not detected in IDP-treated hamster plasma, constituent amino acids of IDPs increased up to 1–2 h and Nπ-methyl-histidine (m-His) remained at high levels up to 6 h after administration. IDP levels in control tissues (vehicle) were similar to those in human tissues. In the IDP group, tissue IDPs were higher than those in the vehicle and m-His increased in all tissues. **Conclusions**: This study indicated that m-His levels increase in hamster tissues following a single oral administration of IDPs and strongly suggest that hamsters should be used in functional studies of IDPs in humans, focusing on the functionality of m-His.

## 1. Introduction

Imidazole dipeptides (IDPs) are endogenous antioxidants synthesized from β-alanine (b-Ala) and L-histidine (His), that is, their synthesis and degradation occur in vivo [1,2]. Carnosine (β-alanyl-L-histidine, Car) is first synthesized by carnosine synthase (Carns-1) and then methylated by carnosine methyltransferase to form anserine (β-alanyl-Nπ-methyl-L-histidine, Ans) [3]. IDPs are abundant in the skeletal muscles of vertebrates, but the types of IDPs present in skeletal muscles differ depending on the species [4]. In humans, Car is predominant. Ans is predominant in mice, rats, dogs, cats, (Ans-to-Car ratio of 10:2 to 3) and chickens (Ans-to-Car ratio of 3:1). In fish, such as tuna and bonito, Ans is predominant, whereas Car is not detected in salmon. However, with improvements in the accuracy of IDP analyses, IDPs other than Car have been detected in trace amounts in human tissue [5].

IDPs exhibit pH-buffering [6], metal-chelating [7], and antioxidant activities [8] that inhibit advanced glycation end product formation [9], prevent neurodegenerative diseases [10], protect tissues from diabetic nephropathy [11], and enhance cardiac function [12]. Therefore, IDPs are considered beneficial substances potent activity for preventing age-related disorders and maintaining overall health. However, many of these studies used animals lacking serum carnosinase (CN1) as test subjects. Therefore, it remained unclear whether the effects of IDPs observed in animal experiments could be replicated in humans, where IDPs are degraded in the blood by CN1. This is because, while oral administration of Ans or Car to mice has been observed to increase levels of Ans and Car respectively in both blood and major tissues [13], in humans, only trace amounts of Ans are detected in the blood, and Car is not detected at all [14].

Despite the above, administration of chicken extract-derived IDPs (Ans/Car = 3/1) reduces oxidative stress in healthy individuals [15]. Additionally, chronic supplementation with the Ans-Car complex derived from chicken extract and Ans derived from salmon extract improves cognitive decline in the elderly and reduces the risk of developing Alzheimer’s disease [16]. These findings suggest that IDPs exert health benefits, particularly as antioxidants, even after being degraded in the blood. IDPs may undergo repeated synthesis and degradation by Carns-1 and carnosinase.

In the blood of primates, such as humans, a carnosine-degrading enzyme called carnosinase 1 (CN1) is always present [17]. However, CN1 is lacking in the blood of other mammals, which only contains carnosinase 2 (CN2), a cytosolic nonspecific dipeptidase, in their tissues. Therefore, in human blood, orally ingested Car is rapidly degraded into b-Ala and His, which are then transported and distributed to various tissues via peripheral circulation. It is hypothesized that Car is resynthesized by Carns-1 in the skeletal muscle, brain, and other tissues, where it exerts various biological functions [4,18,19].

Ans is a methylated form of Car and has a lower affinity for CN1 than for Car, resulting in slower degradation in the human blood following oral administration [20,21]. However, the distribution of Ans in the human body after oral administration remains unclear. Additionally, there is limited knowledge regarding the metabolism of Ans in experimental animals. Therefore, whether the effects of IDPs observed in animal experiments can be replicated in humans is unclear. To the best of our knowledge, no previous studies have used hamsters as model animals for IDP research. In this study, we orally administered IDPs to golden hamsters (*Mesocricetus auratus*), a non-primate mammal that uniquely possesses CN1 in their blood, to investigate the blood levels and distribution of IDPs and related substances in major tissues. This study can help improve the understanding the physiological impact of IDPs in humans, given their diverse biological functions.

## 2. Materials and Methods

### 2.1. Reagents

L-carnosine, L-anserine nitrate, and 3-methyl-L-histidine (Nπ-methyl-L-histidine, m-His) were purchased from Sigma-Aldrich Japan (Tokyo, Japan). The b-Ala, L-histidine (His), trichloroacetic acid, sodium 1-heptanesulfonate, acetonitrile (HPLC grade), and other reagents were purchased from Fujifilm Wako Pure Chemical Corporation (Osaka, Japan). The purified IDPs derived from chicken meat were AC-10(IK)LF (Lot No. 22061301; Tokai Bussan Co. Ltd., Tokyo, Japan), which is a commercially available ingredient in processed foods. It was purified from chicken meat using ion-exchange chromatography and nanofiltration membrane treatment [22] and had a solid Ans/Car content of 91.0%, with a weight ratio of Ans:Car of 2.74:1 (Ans:Car molar ratio of 2.58:1).

### 2.2. Human Plasma

Human plasma samples were prepared from the blood of four healthy middle-aged male volunteers (46.5 ± 4.1 years old) who provided written informed consent. The volunteers fasted overnight and blood was drawn into heparin sodium-containing blood collection tubes. The obtained blood was centrifuged (3000× *g*, 4 °C, 10 min) to collect plasma, which was then stored at −80 °C until the CN1 activity measurement. Although four specimens may not be sufficient for statistical analysis, as shown in the tables and figures, the coefficient of variation (CV) for human data was below 0.2, indicating low inter-individual variability. Therefore, measurements were performed using four specimens in this study.

### 2.3. Administration of IDPs to Hamsters and Collection of Blood and Tissue Samples

Eight-week old male hamsters (Slc: Syrian) were purchased from Japan SLC, Inc. (Shizuoka, Japan) and were acclimatized for 1 week. They were individually housed in plastic cages (W 18.2 × D 26.0 × H 12.8 cm) and provided with solid feed (Labo MR Stock, Nosan Corporation, Tokyo, Japan) and tap water *ad libitum*. The hamsters were randomly divided into two groups of four, fasted for approximately 16 h, and administered IDPs dissolved in water for injections at a dose of 1000 mg/kg body weight (Ans, 733 mg; Car, 267 mg) via forced oral administration (IDP group). The vehicle group was administered with water for injections.

Blood samples were collected sequentially from the jugular vein under isoflurane inhalation anesthesia at time points before IDP administration, and 30 min, 1 h, and 2 h after administration. After 6 h of administration, the abdomen was opened under isoflurane inhalation anesthesia, and blood was collected from the posterior vena cava. Blood was collected into heparin sodium-containing blood collection tubes. The obtained blood samples were centrifuged at 13,600× *g* for 1 min at 4 °C to recover plasma and stored at −80 °C until use. Following blood collection, systemic perfusion was performed using heparin-containing physiological saline solution. The brain, soleus muscle, kidneys, lungs, and liver were harvested and weighed. The tissues were frozen in liquid nitrogen and stored at −80 °C.

### 2.4. Measurement of CN1 Activity

CN1 activity was measured by performing an enzymatic hydrolysis reaction in a 1.5 mL microtube according to the method of Teufel et al. [23], and the residual substrate concentration after the reaction was determined using the HPLC method described below. The reaction mixture (100 μL) contained heparinized human plasma sample (10 μL), 50 mM Tris-HCl buffer (pH 7.5), and 1 mM substrate (Car or Ans). The tubes were pre-warmed at 30 °C in a water bath for 5 min prior to substrate addition. The reaction was initiated by adding the substrate, followed by incubation at 30 °C for 60 min. The reaction was terminated by adding 50 μL of 1% (*w*/*v*) trichloroacetic acid solution to the reaction mixture. For control samples, trichloroacetic acid solution was added before substrate addition. After termination, the mixture was centrifuged at 10,000× *g* at 4 °C for 15 min, and the supernatant was filtered through a disposable filter (pore size 0.2 μm) to obtain the test solution for HPLC. Based on preliminary tests indicating that hamster plasma has higher CN1 activity than that of human plasma, 5 μL of the hamster plasma sample added to the reaction solution and the reaction time was shortened to 5 min.

To calculate the CN1 activity in the sample, the following equation was used:

CN1 activity = (*μmol_CTR_* − *μmol_HYD_*)/(*V* × *t*)

Here, CN1 activity is the plasma hydrolysis rate measured in micro-moles [μmol/(mL × h)] of substrate hydrolyzed per 1 mL of serum over 1 h, *μmol_CTR_* is the substrate concentration determined in the control sample, *μmol_HYD_* is the residual substrate concentration determined in the hydrolysis sample, *V* is the plasma volume (mL), and *t* is the reaction time (h).

### 2.5. Quantification of IDP-Related Compounds

Plasma was obtained by mixing with a cooled trichloroacetic acid solution and centrifuging (10,000× *g*, 15 min, 4 °C) to obtain the supernatant. Each tissue sample was weighed, mixed with a cooled 5% trichloroacetic acid solution, homogenized, and centrifuged as described above to obtain the supernatant. These supernatants were filtered through a disposable filter (pore size 0.2 μm) and used as test solutions. The content of the constituent amino acids of IDPs (b-Ala, His, and m-His) was measured using a Hitachi high-speed amino acid analyzer L-8900 (Hitachi High-Tech Corporation, Tokyo, Japan). Ans and Car contents were measured using the modified reverse-phase ion-pair HPLC-ultraviolet detection method described by Kumagai et al. [24]. The column used was TSK gel ODS-80Ts (150 × 4.6 mm; particle size, 5 μm; Tosoh Corporation, Tokyo, Japan). The HPLC system used was a LC-2030D (Shimadzu, Kyoto, Japan), with a flow rate of 1.0 mL/min, wavelength of 220 nm, and sample injection volume of 10 μL. Buffer A consisted of 50 mM potassium dihydrogen phosphate and 6 mM sodium 1-heptanesulfonate (pH 3.4), whereas buffer B consisted of acetonitrile. The gradient conditions were as follows: 0–4 min: B 0%, 4–12 min: B 4%, 12–17 min: B 40%, and 17–32 min: B 0%. Using this analytical method, it was confirmed that the peaks of Ans and Car did not overlap with those of balenine and homocarnosine.

### 2.6. Protein Degradation Assay for Evaluating Antioxidant Activity Against Hypochlorous Radicals

To evaluate the antioxidant activity of IDPs and related substances, we measured their inhibitory effect on protein degradation by hypochlorous radicals. The measurement of protein degradation inhibition was performed according to the method of Yanai et al. [25]. Briefly, 200 μL of target protein solution (egg white albumin, dissolved in buffered saline at 2.5 mg/mL) was placed in a 1 mL centrifuge tube. Then, 25 μL of each sample solution (final concentration 5 mM) was added and mixed. The mixture was left at room temperature (20–25 °C) for 10 min. Then, 25 μL of hypochlorous acid solution (final concentration 10 mM) was added, and the mixture was incubated at 37 °C for 30 min.

Next, 10 μL of the reaction solution was mixed with an equal volume of 20% 2-mercaptoethanol- and glycerol-containing polyacrylamide gel electrophoresis sample solution (2× concentrated) to stop the reaction. These samples were loaded onto a 10–20% concentration gradient SDS-polyacrylamide gel (Cosmo Bio Co., Ltd., Tokyo, Japan) and electrophoresed at 40 mA for 50 min. After electrophoresis, the gel was stained with Coomassie Brilliant Blue R250 solution (Sigma-Aldrich Japan, Tokyo, Japan) and destained, the band intensity of ovalbumin was quantified using the image processing software ImageJ version 1.54e (National Institutes of Health). The protein degradation inhibition rate for each test substance was calculated using the following formula:

Inhibition rate of protein degradation (%) = (A − B)/(C − B)

where A is the band intensity of the protein treated with the test substance, B is the band intensity of the protein without the test substance, and C is the band intensity of the untreated protein.

### 2.7. Statistical Analysis

Data are presented as mean ± standard error (SE). Differences between groups were evaluated using the free statistical software EZR version 1.68 [26] with Student’s *t*-test or one-way analysis of variance (ANOVA) and Tukey’s multiple comparison test. All tests were considered statistically significant at *p* < 0.05.

## 3. Results

### 3.1. CN1 Activity in Human and Hamster Plasma

To evaluate CN1 activity in hamster plasma, plasma samples were measured and compared with CN1 activity in human plasma (four healthy males, mean age 46.5 ± 4.1 years). CN1 activity in human plasma was 2.73 ± 0.54 μmol/mL/h for Car and 1.08 ± 0.20 μmol/mL/h for Ans. In contrast, CN1 activity in hamster plasma was approximately 20 times higher for Car and approximately 12 times higher for Ans (53.1 ± 1.8 μmol/mL/h and 12.9 ± 0.4 μmol/mL/h, respectively) compared with those in human plasma (Table 1).

### 3.2. Changes in the Concentration of IDP-Related Compounds in Hamster Plasma After IDP Administration

**Figure 1:**
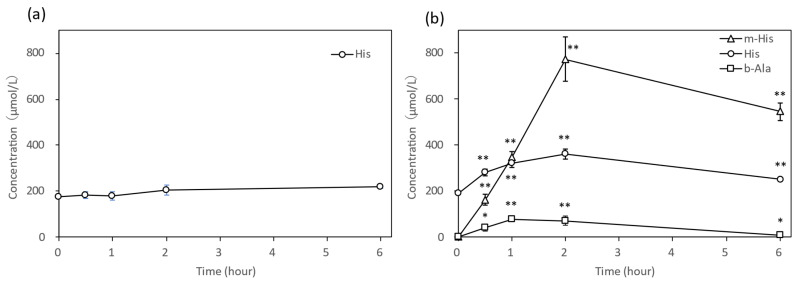
Concentration changes in IDP-related compounds in hamster plasma after administration of water for injection (**a**) or IDPs 1000 mg/kg body weight (**b**) (μmol/L, n = 4). Circles, His; triangles, m-His; squares, b-Ala. * Significant difference from time 0: * *p* < 0.05, ** *p* < 0.01. b-Ala, β-alanine; His, L-histidine; IDP, imidazole dipeptide; m-His, Nπ-methyl-histidine.

After administering IDPs at a dose of 1000 mg/kg body weight via forced oral administration to hamsters, we investigated the plasma concentration profiles of IDP-related compounds for up to 6 h after administration. In the vehicle group, neither Ans, Car, m-His, nor b-Ala were detected in the plasma; only His was detected (Figure 1a). Conversely, in the plasma of the IDP group, Ans and Car were not detected at 6 h after administration, but b-Ala, His, and m-His, which are the constituent amino acids of Car and Ans, were detected 30 min after administration (Figure 1b). Furthermore, b-Ala and His returned to pre-administration levels at 6 h, whereas m-His maintained a high concentration (545.5 ± 37.9 μmol/L) even 6 h after administration.

### 3.3. Contents of IDP-Related Compounds in Hamster Tissues 6 H After IDP Administration

**Figure 2:**
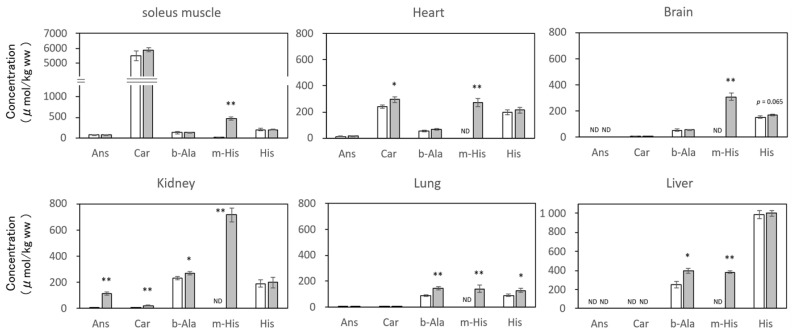
Concentration of IDP-related compounds in hamster tissues 6 h after IDP administration (unit: μmol/kg, n = 4 in each group). White bars: control group; gray bars: IDP group. Significant differences from the control group by *t*-test: * *p* < 0.05; ** *p* < 0.01; ND, not detected. Ans, anserine; b-Ala, β-alanine; Car, carnosine; His, L-histidine; IDP, imidazole dipeptide; m-His, Nπ-methyl-histidine.

As described above, neither Ans nor Car were detected in hamster plasma up to 6 h after IDP administration, and only their constituent amino acids were detected (Figure 1b). On the other hand, in hamster tissues, Car was present at high concentrations in the soleus muscle and heart without IDP administration, while Ans was present in extremely trace amounts (Figure 2). In other tissues, both Car and Ans were present only at trace levels. Furthermore, b-Ala and His, the constituent amino acids of Car, were present in all tissues, but m-His, a constituent of Ans, was not detected in all tissues except the soleus muscle. In contrast, m-His was detected at significantly high concentrations in all tissues 6 h after IDP administration. The m-His concentrations in the soleus muscle and kidneys were particularly high, followed by the liver, brain, heart, and lungs in descending order. The levels of b-Ala and His, constituent amino acids of Car, slightly increased in all tissues compared with those of m-His. Notably, in the soleus muscle and heart, where Ans and Car are typically present, increases in Ans and Car levels were observed; in particular, Car levels significantly increased in the heart (*p* < 0.05). Although at trace levels, significant increases in Ans and Car were detected in the kidneys (both *p* < 0.01), whereas no increases in IDP levels were detected in the brain or lungs; however, m-His, b-Ala, and His were significantly increased as well in kidneys. Other IDP constituent amino acids, b-Ala and His, increased in all tissues following IDP administration, with the most pronounced increases in b-Ala observed in the lungs, liver, and kidneys (*p* < 0.01, *p* < 0.05, and *p* < 0.05, respectively).

**Figure 3:**
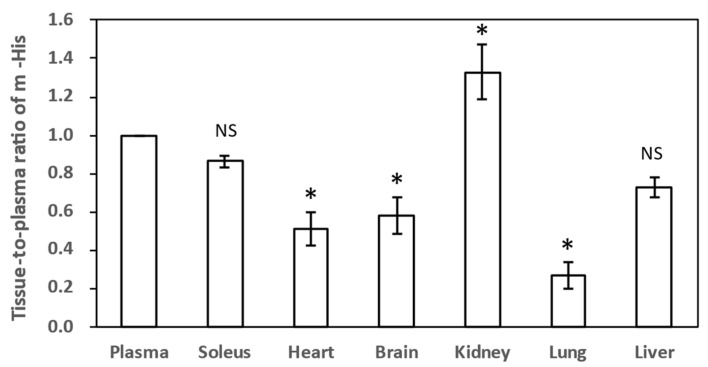
The tissue-to-plasma concentration ratio of m-His at 6 h after IDP administration (n = 4). The plasma concentration of m-His 6 h after IDP administration was 545 ± 38 μmol/L. * Significant differences from the mean plasma value were observed using the Tukey multiple comparison test (*p* < 0.05). NS, not significant; IDP, imidazole dipeptide; m-His, Nπ-methyl-histidine; Soleus, soleus muscle.

In the vehicle group, m-His, a constituent amino acid of Ans, was detected only in trace amounts in the soleus muscle but was below the detection limit in other tissues. However, following IDP administration, the m-His concentration in each tissue significantly increased, which was attributed to the distribution of m-His generated in plasma to each tissue. Therefore, we summarized the tissue transfer rate of m-His based on the concentration ratios of m-His in each tissue and plasma sample 6 h after IDP administration (Figure 3). In the kidneys, m-His was not detectable before IDP administration (control group); however, 6 h later, it was 1.33 times higher than that in plasma, indicating the accumulation of m-His. Subsequently, the tissue-to-plasma concentration ratio in the soleus muscle was 0.85, while that in the liver, heart, and brain was 0.55–0.75, which was comparable to that in the soleus muscle. The tissue-to-plasma concentration ratio in the lungs was only 0.3.

### 3.4. Antioxidant Activity of IDP-Related Compounds Against Hypochlorous Radicals

**Figure 4:**
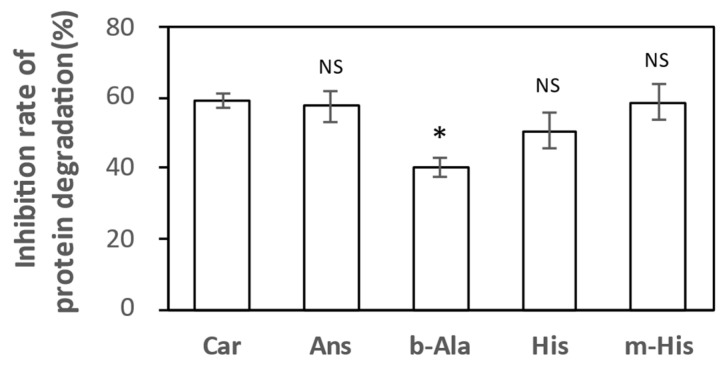
Inhibitory effects of Ans-Car-related compounds on protein degradation by hypochlorous radical. The values indicate inhibition rates of protein degradation (n = 5). * A significant difference was observed between the mean inhibition rate of Car using Tukey’s multiple comparison test (*p* < 0.05). NS, not significant; Ans, anserine; b-Ala, β-alanine; Car, carnosine; His, L-histidine; m-His, Nπ-methyl-histidine.

In our previous study [25], we found that IDPs and amino acids exhibit specific antioxidant activity against hypochlorous radicals among three endogenous reactive oxygen species (hydroxyl, hypochlorous, and peroxynitrite radicals). In this study, we tested how strong the antioxidant activity of IDP constituent amino acids was compared to Car and Ans. As shown in Figure 4, the antioxidant activity measured by the inhibitory effect of m-His on protein-degradation by hypochlorous radicals was nearly equivalent to that of Car and Ans. His was slightly weaker than m-His but stronger than b-Ala. b-Ala was significantly lower than Ans, Car, and m-His. This finding suggests that m-His may possess antioxidant activity comparable to that of IDPs.

## 4. Discussion

Experiments involving the oral administration of IDPs to animals have been conducted to investigate their physiological functions in vivo. However, the IDP-degrading enzyme CN1 is present in the blood of primates such as humans, causing IDPs to be rapidly degraded into their constituent amino acids during absorption from the intestinal tract and entry into the blood. In contrast, CN1 is absent from the blood of non-primate mammals, meaning that administered IDPs are distributed throughout the body without degradation [13]. Therefore, when investigating the in vivo function of orally administered IDPs in non-primate animals, there is concern about whether the results from animal studies can be directly extrapolated to humans.

Among non-primate mammals, only the golden hamster has CN1 activity in its blood, which is stronger than that found in humans [17]. In our study, CN1 activity in hamster plasma was approximately 20 times higher for Car and around 12 times higher for Ans than that in healthy humans (Table 1). As these results reflect the effect of CN1 in the blood, it was thought that hamsters are considered the most appropriate model for functional studies involving the oral administration of IDPs and extrapolation to humans. Since no studies using hamsters as model animals for IDP research have been reported, in this study, we orally administered IDPs to hamsters and investigated their distribution in the blood and other major tissues.

**Figure 5:**
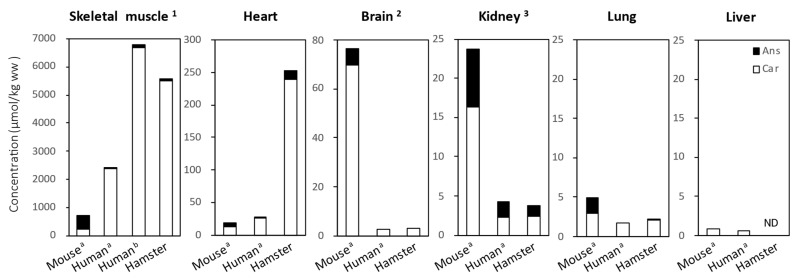
Comparison of anserine and carnosine content (μmol/kg wet weight) in hamster tissues with values reported in the literature for mice and humans. Black bars: anserine; White bars: carnosine. a, Values reported by Van der Stede et al. [5] (modified and reprinted with permission from John Wiley and Sons, Inc.). b, Values reported by Hoetker et al. [27]. 1, Mice and hamsters: soleus muscle; humans: lateral vastus muscle. 2, Mice and humans: cerebral cortex (white matter); hamsters: entire brain. 3, Mice and hamsters: entire kidneys; humans: kidneys (medulla). ND, not detected; Ans, anserine; Car, carnosine.

Previous studies have reported IDP levels in mouse and human tissues. Therefore, we measured the IDP levels in hamster tissues without administering IDPs and compared them with the previously reported values in mice and humans (Figure 5).

According to Van der Stede et al. [5] and Hoetker et al. [27], although IDP levels are lower in mice than in humans, the Ans:Car ratio in mouse skeletal muscles significantly differs from that in humans. In the mouse soleus muscle, Ans is dominant and the Ans:Car ratio is 1.87/1 [5], whereas in the human lateral vastus muscle, Car is dominant and the Ans:Car ratio is 1/68.5 [5] or 1/60.2 [27]. In contrast, Car is dominant in the soleus muscle of hamsters, with an Ans:Car ratio of 1/65.4, similar to that in humans. Furthermore, the total IDP content in the soleus muscle of hamsters is 5587 ± 349 μmol/kg ww and is comparable to that in the human lateral vastus muscle (6791 ± 206 μmol/kg ww) [27].

In hamster hearts, the Ans:Car ratio (1/17.4) is closer to that in humans (1/21.0) [5] rather than that in mice (1/2.8) [5]; however, the total IDP content (253.6 ± 13.2 μmol/kg ww) is approximately 10 times higher than those of mice and humans (18.4 ± 8.6 μmol/kg ww and 27.7 ± 22.5 μmol/kg ww, respectively [5]). In hamster brains, only Car was detected at 3.26 ± 1.44 μmol/kg ww, which was nearly identical to the levels in the human cerebral cortex (2.56 ± 1.77 μmol/kg ww) [5]. In hamster kidneys and lungs, both the total IDP content and Ans:Car concentration ratio were closer to those in humans than to those in mice. In the liver, although Car was reported to be present only in humans and mice, neither Ans nor Car was detected in hamsters.

Notably, the ratio and total content of IDPs in hamster tissues were more similar to those in humans than to those in mice (Figure 5). In this study, we did not investigate the expression levels of *CARNS1* in hamster tissues; however, its expression levels in humans, mice, and rats are proportional to the tissue IDP content at the systemic level [5]. Based on these findings, despite differences in the intensity of CN1 activity in the plasma, both the expression levels of *CARNS1* and tissue IDPs in various tissues may be similar between hamsters and humans.

**Figure 6:**
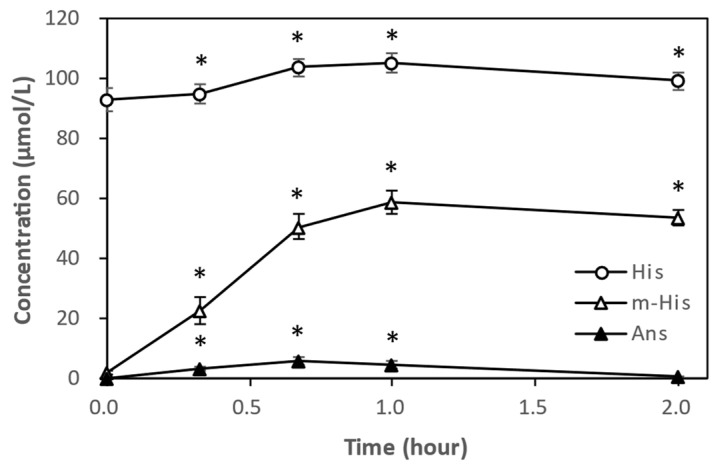
Changes in plasma concentrations of IDP-related compounds in healthy middle-aged men after oral administration of 1000 mg of IDPs (μmol/L, n = 10). Modified from the report by Shiotani et al. [14]. White circles: His; white triangles: m-His; filled triangles: Ans. * Significant difference from time 0 (*p* < 0.01). Ans, anserine; His, L-histidine; IDP, imidazole dipeptide; m-His, Nπ-methyl-histidine.

Following IDP administration, only b-Ala, m-His, and His were detected in the plasma of hamsters. Furthermore, m-His remained at high concentrations in the plasma even 6 h after IDP administration (Figure 1). Due to the infrequent measurement intervals for m-His concentration in plasma, precise C_max_ or plasma half-life could not be determined. However, results from our previous human trials [14] suggested that C_max_ appears between 1 and 2 h (Figure 6). Based on the decreasing trend observed from 2 to 6 h, the plasma half-life of m-His was estimated to be 9 h (Figure 3). Furthermore, while neither Ans nor Car were detected in hamsters 30 min after IDP administration, Ans was detected in human plasma with weak CN1 activity from immediately after administration up to 1 h. Car was not detected from immediately after administration, similar to hamsters. This plasma concentration profile of IDPs was thought to reflect the strength of plasma CN1 activity.

The concentration ratios in plasma and various tissues indicate the transfer of m-His from plasma to each tissue, with the highest transfer occurring to the kidneys (Figure 3). The transfer rates to the soleus muscle and liver were also high, at 0.80–0.90. In contrast, the brain, heart, and lungs showed transfer rates of approximately 0.40–0.50 of the plasma level.

Kubomura et al. orally administered tuna-derived Ans to healthy subjects and reported a plasma half-life of 7.3 h for m-His [28]. In addition, it has also been reported that m-His may serve as a biomarker for chicken and animal meats such as beef or pork consumption [29]. The key points here are that in humans possessing CN1 activity, among the IDP components that could potentially substitute for IDPs, b-Ala is preferentially utilized in other metabolic pathways such as transamination and energy supply [30], and His is not only a precursor to carnitine but is also essential for protein synthesis; excess amounts are converted into other substances such as histamine and catabolizes [31]. In contrast, m-His possesses a relatively long half-life and is not utilized as a substrate for protein synthesis, strongly suggesting its potential to substitute for the action of IDPs in vivo.

In addition, we were unable to measure urinary excretion after IDP administration in this study; thus, we discuss this point based on prior studies. Abe et al. [32] reported that when humans ingested IDPs derived from tuna meat (Ans content 90%), Ans and m-His were rapidly excreted in urine. They reported that the urinary excretion rates for Ans and m-His were 8% and 80%, respectively. In contrast, for eel-derived IDPs primarily composed of Car (Car content 90%), Car excretion was only 1%. Furthermore, they reported that the peak urinary concentration of IDPs and related substances was reached at 7 h, and they completely disappeared from urine 40 h after ingestion. From this finding, it was speculated that m-His might be accumulated in tissues, although not done in this study, the influence of m-His accumulation in tissues should be elucidated in future studies.

Various biochemical properties and physiological functions of IDPs have been reported; however, reports on m-His are limited. m-His exhibits copper ion-chelating activity equivalent to or greater than that of Car and Ans [33]. In vitro studies have demonstrated that IDPs and m-His may reduce serum uric acid levels by inhibiting uric acid uptake by renal cells and suppressing xanthine oxidase activity [34]. Additionally, m-His and Ans were recently reported to restore the loss of electrical resistance caused by methylglyoxal in epithelial and endothelial cells [35].

Incidentally, human studies have reported that sustained supplementation with chicken extract-derived IDPs (Ans/Car = 3/1) or salmon-derived Ans alone improved cognitive function in the elderly and reduced the risk of Alzheimer’s disease [36,37]. Consequently, it has been reported that m-His and His concentrations in the cerebrospinal fluid of high-risk groups for Alzheimer’s disease are lower than those in healthy individuals with normal cognitive function [38]. Furthermore, as shown in Figure 2, it was remarkable event that the concentration of m-His after IDP administration significantly increased in all tissues examined. Although currently no reports whether m-His crosses the blood–brain barrier, it is presumed that the increase in m-His in the brain may be due to its accumulation from the bloodstream through the blood–brain barrier, as well as His [39]. On the other hand, the increases in IDPs, b-Ala, and His were lower than those observed for m-His. This suggests that m-His instead of IDPs may have a suppressive action against damage caused by reactive oxygen species in all tissues, not limited to the brain or skeletal muscle. Particularly, due to its notable transfer to the kidneys, m-His generated from administration of Ans may related to potentially beneficial effect of IDPs shown in animal studies for preventing kidney disease [39,40]. In this study, although m-His revealed the same antioxidant activity of IDPs, it was not clear if m-His could substitute IDPs in hamster possessing CN1. Further study will be needed.

Other findings in this study include the observation of an increase in Car in hamster hearts and soleus muscles. The reason for this remains unclear. Whether Car from orally administered IDPs migrated without degradation [41] or whether Car synthesis occurred in these tissues should be elucidated in future studies.

## 5. Conclusions

Although the findings of this study are limited, oral administration of IDPs to hamsters, which, like humans, possess CN1 in their blood, resulted in a rapid increase in m-His—a constituent amino acid of Ans—rather than IDPs themselves in the blood. This is in stark contrast to animals lacking CN1, where IDPs increase. In humans, who possess approximately 1/20 of the CN1 activity of hamsters, trace amounts of Ans were detected, but the blood profile resembled that of humans, with m-His maintaining high concentrations rather than IDPs. Following IDP administration, m-His distributed throughout systemic tissues, accumulating at high concentrations particularly in skeletal muscle, kidneys, brain, and heart. These findings strongly suggest that hamsters should be used in functional studies of IDPs in humans, focusing on the functionality of m-His.

## Figures and Tables

**Table 1:** CN1 activity in human and hamster plasma.

Substrate	Degradation Activity (μmol/mL/h) ^1^		*p* Value (Human vs. Hamster)	Activity Ratio (Hamster/Human)
	Human	Hamster		
Carnosine	2.73 ± 0.54	53.1 ± 1.8	<0.001	19.5
Anserine	1.08 ± 0.20	12.9 ± 0.4	<0.001	11.9

^1^ The average values ± standard error of plasma CN1 activity were obtained from four male subjects (aged 46.5 ± 4.1 years) and four male hamsters (8 weeks old).

## Data Availability

The original contributions presented in this study are included in the article. Further inquiries can be directed to the corresponding author.

## Abbreviations

The following abbreviations are used in this manuscript:

Ans	L-anserine
b-Ala	β-alanine
Car	L-carnosine
Carns-1	carnosine synthase
CN1	serum carnosinase
IDP	Imidazole dipeptide
His	L-histidine
m-His	1-methyl-histidine (Nπ-methyl-L-histidine)
